# Needle Track Seeding of Primary and Secondary Liver Carcinoma After Percutaneous Liver Biopsy

**DOI:** 10.1155/1993/39539

**Published:** 1993

**Authors:** Timothy G. John, O. James Garden

**Affiliations:** University Department of Surgery The Royal Infirmary Lauriston Place Edinburgh EH3 9YW UK

## Abstract

Seeding of tumour in the needle track following percutaneous needle biopsy of liver neoplasms is rarely reported. We describe two such cases following the needle biopsy of an hepatocellular carcinoma and secondary colorectal carcinoma respectively. The risk of needle track recurrence of liver tumours should not be regarded as insignificant. The diagnosis of liver neoplasms may be achieved by non-invasive modalities, and their needle biopsy should be reserved for cases not amenable to surgical resection.


*HPB Surgery*, 1993, Vol. 6, pp. 199–204
Reprints available directly from the publisher
Photocopying permitted by license only

© 1993 Harwood Academic Publishers GmbH
Printed in the United States of America

### CASE REPORT

### NEEDLE TRACK SEEDING OF PRIMARY AND SECONDARY LIVER CARCINOMA AFTER PERCUTANEOUS LIVER BIOPSY

TIMOTHY G. JOHN and O. JAMES GARDEN
*University Department of Surgery, The Royal Infirmary, Edinburgh*

*(Received 17 December 1991)*

Seeding of tumour in the needle track following percutaneous needle biopsy of liver neoplasms is rarely reported. We describe two such cases following the needle biopsy of an hepatocellular carcinoma and secondary colorectal carcinoma respectively. The risk of needle track recurrence of liver tumours should not be regarded as insignificant. The diagnosis of liver neoplasms may be achieved by non-invasive modalities, and their needle biopsy should be reserved for cases not amenable to surgical resection.

KEY WORDS: Neoplasm seeding, biopsy, needle, liver neoplasm

### INTRODUCTION

Percutaneous needle biopsy is a commonly used technique in the diagnosis of malignant abdominal tumours, and it is widely held that the advantage of a high diagnostic yield outweighs the low complication rate. In particular, the recurrence of tumour in the biopsy track is said to be rare, with seeding frequencies of 0.005–0.009% quoted for large series of fine-needle abdominal biopsies[1,2]. However, as isolated cases of needle track seeding continue to be reported, there have been calls for caution in view of this real, albeit small, risk. We report two cases where local dissemination of primary and secondary liver carcinomas occurred following percutaneous needle biopsy.

### CASE REPORTS

### *Case 1*

A 71 year old male presented with epigastric pain, anorexia and weight loss, and examination revealed irregular hepatomegaly. A 10 cm mass occupying the left lobe of the liver was seen on ultrasound scan. Serum $\gamma$-glutamyl transferase and

Address correspondence to: Mr T.G. John, University Department of Surgery, The Royal Infirmary, Lauriston Place, Edinburgh EH3 9YW, UK

alkaline phosphatase were elevated at 313 μ/l (normal range 0–45 μ/l) and 373 μ/l (normal range 60–260 μ/l) respectively. Serum α-fetoprotein was markedly raised at 14 700 μ/l (normal range 0–15 μ/l).

A Trucut® (Travenol Laboratories Inc., Illinois, USA) liver biopsy was attempted and a single core of normal subcapsular liver tissue obtained. An ultrasound - guided needle biopsy using two passes through the left upper quadrant of the abdomen with an 18G Surecut® needle yielded tissue reported as poorly differentiated hepatocellular carcinoma. The patient was referred for consideration of hepatic resection.

An iodised oil emulsion (IOE) enhanced CT scan and hepatic angiography demonstrated a vascular, lobulated tumour, arising in segments 2 and 3, and extending into segment 4. No extrahepatic tumour extension was demonstrated on CT scanning of the abdomen and thorax, and the tumour was considered suitable for left hemihepatectomy. At laparotomy, two months after initial presentation, a 2 × 0.5 cm hard nodule was discovered in the left rectus muscle with several further nodules in the underlying falciform ligament. This coincided with the site of the repeat needle biopsy and histology confirmed metastatic hepatocellular carcinoma. Operative ultrasonography demonstrated tumour confined to the left hemiliver with the middle hepatic vein free of tumour. There was no evidence of further peritoneal dissemination, but no resection was undertaken in view of the local tumour spread. Hepatic arterial embolization with Lipiodol and Adriamycin was performed one month later but not tolerated well, and further intervention was deemed inappropriate. The patient died three months later from locally invasive tumour.

*Case 2*

A 59 year old female presented in September 1989 with malaise, anorexia and weight loss, and was found to have four fingerbreadth hepatomegaly. She had a past history in 1983 of resection of a tubulovillous adenoma of the sigmoid colon. Histology had indicated marked glandular dedifferentiation with an area of early invasion of the muscularis mucosa. Serial barium enemas and colonoscopies were normal until January 1989 when a further tubulovillous adenoma of the rectum was excised. An ultrasound and CT scan demonstrated a solitary mass confined to the right hemiliver. A Trucut biopsy of the liver lesion under ultrasound guidance was obtained at this stage, the histological appearances of which suggested a secondary adenocarcinoma and the patient was referred for assessment for hepatic resection.

Hepatitis B serology was negative and serum α-fetoprotein levels normal at 2 μ/l (normal range 2–6 μ/l). Serum carcino embryonic antigen (CEA) levels were profoundly elevated at 40 800 μ/l (normal range 0–35 μ/l). An IOE enhanced CT scan demonstrated a well defined tumour mass involving segments 1,4,5 and 8. The tumour was judged to be at the limits of resectability, and a course of chemoembolisation was undertaken to provide both symptomatic relief and reduction in tumour volume sufficient to enable hepatic resection. Three hepatic arterial embolizations with Adriamycin, Lipiodol and Gelfoam were well tolerated, with good palliation of symptoms. However, serial CT scans demonstrated no significant change in tumour size, with serum CEA levels rising to 167 000 μ/l over three months. She was readmitted in September 1990 with intractable pain which appeared to be arising from a tender nodule situated over the right eighth rib laterally at the site of the percutaneous liver biopsy performed 12 months before. Two discrete hard 1 cm subcutaneous nodules were biopsied and histology revealed a well differentiated adenocarcinoma, strongly suggestive of a colonic metastasis. The patient died two months later from local progression of her tumour.

### DISCUSSION

Review of the literature has revealed seven previous cases of local recurrence of hepatocellular carcinoma in a percutaneous needle biopsy track[1,3–7], and two instances of needle track seeding of secondary liver tumours[8,9]; these are summarised in the table. Three cases followed "curative" resection of hepatocellular carcinomas at 8 month - 4 year intervals[3,5]. Our first case is noteworthy in that the needle track dissemination of hepatocellular carcinoma was evident at laparotomy within two months of biopsy, and as with another reported case[7], the excision of an otherwise resectable tumour was precluded. In both cases the biopsies were unnecessary for diagnosis, as the nature of the malignancy was strongly suggested by radiological imaging and gross elevation in tumour markers. Both previously reported cases of recurrence of secondary colonic carcinoma in percutaneous needle tracks were in patients who had previously undergone hepatic resection, but who had developed recurrent liver tumour which had been biopsied. In another case[9], extrahepatic tumour recurrence of a secondary rectal carcinoma occurred 13 months following intraoperative direct needle biopsy and wide resection of a deep-seated solitary liver metastasis, and was attributed to spillage of malignant cells at the time of biopsy.

It is generally accepted that percutaneous needle biopsy combined with radiological imaging is a safe and accurate means of confirming a tissue diagnosis in focal neoplastic liver disease[10,11]. The apparent rarity of malignant implantation from abdominal neoplasms has been emphasised in large series of fine-needle aspiration biopsies[1,2,12], by definition using a needle of 1 mm diameter (19 gauge) or less, although seeding of pancreatic[13–16] and renal cell carcinoma[17] has been described following this technique. Conventional core-cutting needle biopsy is assumed to carry an increased risk of seeding, and this has been documented for carcinoma of the prostate[18], lung[19] and thyroid[20]. There is no direct evidence that core-cutting needles significantly increase the risk of tumour seeding, however, and it has been demonstrated in animal models that it is possible to implant $10^3$–$10^5$ tumour cells in the tracks of fine-needle aspiration biopsies[21]. Review of the cases where needle track seeding of liver tumours has occurred reveals no specific association with core-cutting needles (see Table 1).

The risk of seeding following needle biopsy of liver tumours can no longer be regarded as insignificant, and this case report provides further evidence that percutaneous needle biopsy track seeding of liver carcinomas may not be as rare as has been claimed. Its true incidence is probably under-reported in that many patients in whom a needle biopsy diagnosis of hepatic malignancy is obtained will succumb with terminal carcinomatosis before metastasis to the parietes becomes apparent, although there is no evidence that biopsy of liver tumour adversely affects survival.

Hepatic resection for hepatocellular carcinoma[22] and colorectal metastases[23] in

**Table 1** Liver tumour seeding following percutaneous needle biopsy: reported cases

| *Patient details* | *Needle type* | *Interval** | *Ref. No.* |
|---|---|---|---|
| N/A | 22G (aspiration) | 2 months | 1 |
| 62 yrs male (HCC) | Trucut (core-cutting) | 8 months | 3 |
| 48 yrs female (HCC) | 22G (aspiration) | 3 months | 4 |
| 70 yrs male (HCC) | Trucut (core-cutting) | 12 months | 5 |
| 64 yrs male (HCC) | N/A | 4 years | 5 |
| 52 yrs female (HCC) | 22G (aspiration) | 3 years | 6 |
| 40 yrs male (HCC) | Trucut (core-cutting) | 3 weeks | 7 |
| 50 yrs female (colonic metastasis) | 20–23G (aspiration) | 4 months | 8 |
| 74 yrs male (colonic metastasis) | 'Thin-needle biopsy' | 17 days | 9 |
| 71 yrs male (HCC) | 18G Surecut (core-cutting) | 2 months | Current |
| 59 yrs female (colonic metastasis) | Trucut (core-cutting) | 1 year | Current |

HCC = Hepatocellular carcinoma, N/A = Details not available
* Time interval between liver biopsy and presentation with needle track tumour dissemination.

selected cases now carries low morbidity and mortality rates, and for some offers the chance of cure. The resectability of hepatic tumours may be assessed by the non-invasive techniques of ultrasound and contrast-enhanced CT scanning, augmented by hepatic angiography, and early biopsy in the management of focal liver lesions can usually be avoided[24]. Percutaneous needle biopsy is only indicated to obtain an histopathological diagnosis on which to make a treatment decision when the tumour is irresectable, or to confirm cirrhosis, or other condition precluding resection, in tumour-free liver.

### *References*

1. Smith, H.E. (1991) Complications of percutaneous abdominal fine-needle biopsy. *Radiology*, **178**, 253–258
2. Livraghi, T., Damascelli, B., Lombardi, C. and Spagnoli, I. (1983) Risk in fine-needle abdominal biopsy. *Journal of Clinical Ultrasound*, **11**, 77–81
3. Sakurai, M., Seki, K., Okamura, J. and Kuroda, C. (1983) Needle tract implantation of hepatocellular carcinoma after percutaneous liver biopsy. *American Journal of Surgical Pathology*, **7**, 191–195
4. Onodera, H., Oikawa, M., Abe, M., Chida, N., Kimura, S., Satake, K., Motojima, T. and Goto, Y. (1987) Cutaneous seeding of hepatocellular carcinoma after fine-needle aspiration biopsy. *Journal of Ultrasound in Medicine*, **6**, 273–275
5. Evans, G.H.C., Harries, S.A. and Hobbs, K.E.F. (1987) Safety of and necessity for needle biopsy of liver tumours (letter). *Lancet*, **i**, 620
6. Tabata, T., Usami, M., Ohmiya, H., Ohyanagi, H., Saitoh, Y., Ueda, K. and Tomita, K. (1990) A case report of cutaneous seeding of hepatocellular carcinoma following ultrasound guided fine needle aspiration biopsy. *Nippon Shokakibyo Gakkai Zasshi*, **87**, 1253–1257
7. Quaghebeur, G., Thompson, J.N., Blumgart, L.H. and Benjamin, I.S. (1991) Implantation of hepatocellular carcinoma after percutaneous needle biopsy. *Journal of the Royal College of Surgeons of Edinburgh*, **36**, 127
8. Glaser, K.S., Weger, A.R., Schmid, K.W. and Bodner, E. (1989) Is fine-needle aspiration of tumours harmless (letter)? *Lancet*, **i**, 620
9. Scheele, J. and Altendorf-Hofman, A. (1990) Tumor implantation from needle biopsy of hepatic metastases. *Hepatogastroenterology*, **37**, 335–337

10. Schwerk, W.B. and Schmitz-Moormann, P. (1981) Ultrasonically guided fine-needle biopsies in neoplastic liver disease. *Cancer*, **48**, 1469–1477
11. Ho, C.S., McLoughlin, M.J., Tao, L.C., Blendis, L. and Evans, W.K. (1981) Guided percutaneous fine-needle aspiration biopsy of the liver. *Cancer*, **47**, 1781–1785
12. Kline, T.S. and Neal, H.S. (1978) Needle aspiration biopsy: critical appraisal. Eight years and 3<ts>267 specimens later. *Journal of the American Medical Association*, **239**, 36–39
13. Ferrucci, J.T., Wittenberg, J., Margolies, M.N. and Carey, R.W. (1979) Malignant seeding of the tract after thin-needle aspiration biopsy. *Radiology*, **130**, 345–346
14. Smith, F.P., MacDonald, J.S., Schein, P.S. and Ornitz, R.D. (1980) Cutaneous seeding of pancreatic cancer by skinny-needle aspiration biopsy. *Archives of Internal Medicine*, **140**, 855
15. Caturelli, E., Rapaccini, G.L., Anti, M., Fabiano, A. and Fedeli, G. (1985) Malignant seeding after fine-needle aspiration biopsy of the pancreas. *Diagnostic Imaging in Clinical Medicine*, **54**, 88
16. Rashleigh-Belcher, H.J.C., Russell, R.C.G. and Lees, W.R. (1986) Cutaneous seeding of pancreatic carcinoma by fine-needle aspiration biopsy. *British Journal of Radiology*, **59**, 182–183
17. Bush, W.H., Burnett, L.L. and Gibbons, R.P. (1977) Needle track seeding of renal cell carcinoma. *American Journal of Roentgenology*, **129**, 725–727
18. Desai, S.G. and Woodruff, L.M. (1974) Carcinoma of prostate. Local extension following perineal needle biopsy. *Urology*, **3**, 87–88
19. Berger, R.L., Dargan, E.L. and Huang, B.L. (1972) Dissemination of cancer cells by needle biopsy of the lung. *Journal of Thoracic and Cardiovascular Surgery*, **63**, 430–432
20. Crile, G. and Hazard, J.B. (1951) Classification of thyroiditis, with special reference to the use of needle biopsy. *Journal of Clinical Endocrinology*, **11**, 1123–1127
21. Ryd, W., Hagmar, B. and Eriksson, O. (1983) Local tumour cell seeding by fine-needle aspiration biopsy: a semiquantitative study. *Acta Pathalogica Microbiologica Immunologica Scandanavica*, **91**, 17–21
22. Adson, M.A. and Weiland, L.H. (1981) Resection of primary solid hepatic tumours. *American Journal of Surgery*, **141**, 18–21
23. Scheele, J., Stangl, R. and Altendorf-Hoffmann, A. (1990) Hepatic metastases from colorectal carcinoma: impact of surgical resection on the natural history. *British Journal of Surgery*, **77**, 1241–1246
24. Thompson, J.N., Gibson, R., Czerniak, A. and Blumgart, L.H. (1985) Focal liver lesions: a plan for management. *British Medical Journal*, **290**, 1643–1645

(*Accepted by S. Bengmark 6 February 1992*)

### INVITED COMMENTARY

This paper clearly demonstrates yet again that no clinical investigation is without risk. For many years it has been taught by the hepatologists that any space occupying lesion in the liver should be subjected to percutaneous biopsy to ascertain its nature. Several of us have urged caution in carrying out this procedure and this paper reinforces this view since an otherwise curable cancer may be disseminated by such a technique. It has been argued that fine needle aspiration will reduce this risk and this is probably true but a risk remains.

I think it is very important to insist that apart from academic studies conducted within strict protocols, no investigation, especially an invasive one, be carried out unless it can be demonstrated that the result will affect subsequent management. In the case of a space occupying lesion in the liver, there are available a multitude of investigative procedures, as a result of which a fairly certain diagnosis, especially in relation to its benign or malignant nature, can be made. Once a lesion has been identified and there is a strong suspicion that it is malignant or if it is causing symptoms, then the question of surgical resection must be considered. Resection of lesions from otherwise normal livers in major centres is an acceptable procedure accompanied by a low morbidity and mortality. However this is not so if the unaffected liver is cirrhotic. If there is clinical evidence to suggest this might be the case then a needle biopsy of the unaffected liver should be done to confirm or refute this. If the liver is normal then it is safe to proceed with resection of the symptomatic or suspicious space occupying lesion without accurate histological diagnosis in anticipation of total cure. If the liver is cirrhotic then histological confirmation of the nature of the pathology of the lesion may be fully justified before considering surgery since the risk of seeding in these circumstances has to be matched against the risk of unnecessary surgery.

Let us hope that the message clearly expressed in this article will be appreciated, especially by our medical colleagues who often undertake needle biopsy of intrahepatic lesions before referring a patient for a surgical opinion.

K.E.F. Hobbs
Professor of Surgery

### INVITED COMMENTARY

This paper clearly documents two further patients in whom an unnecessary percutaneous needle biopsy of an hepatic tumour has resulted in needle track seeding. Such biopsies of potentially resectable hepatic lesions are hardly ever indicated, and may, as in these patients, preclude a later potentially curative hepatic resection. An increased awareness of this uncommon, but probably under-reported, complication of percutaneous biopsy should help to reduce the incidence of this problem.

J.N. Thompson
Senior Lecturer
Hon. Consultant Surgeon
Royal Postgraduate Medical School
Hammersmith Hospital
London W12 0NN

